# Ducks’ Growth, Meat Quality, Bone Strength, and Jejunum Strength Depend on Zeolite in Feed and Long-Term Factors

**DOI:** 10.3390/ani11041015

**Published:** 2021-04-03

**Authors:** Jakub Biesek, Mirosław Banaszak, Marek Adamski

**Affiliations:** Department of Animal Breeding and Nutrition, Faculty of Animal Breeding and Biology, UTP—University of Science and Technology in Bydgoszcz, Mazowiecka 28, 85-084 Bydgoszcz, Poland; miroslaw.banaszak@utp.edu.pl (M.B.); adamski@utp.edu.pl (M.A.)

**Keywords:** age, aluminosilicate, bones, duck, gut, meat traits, origin, rearing, sex, strength

## Abstract

**Simple Summary:**

Natural agents ensuring the biosecurity of poultry production and the high quality of meat have again gained increasing interest. Natural zeolites absorb toxic gases and can also stimulate digestion and improve the physicochemical parameters of meat, which is important from the consumer’s point of view. The aim of the study was to assess the effect of a diet with a 4% inclusion of zeolite on the growth, the meat quality, and the strength of the bones and jejunum of Orvia and Cherry Valley ducks of different age and sex. The addition of zeolite was associated with reduced body weight gains and an increased feed conversion ratio, but with a higher water-holding capacity of breast muscles in seven-week-old ducks, and with a higher yellowness and water-holding capacity of leg muscles in six-week-old ducks. Interactions were found between long-term factors (age, sex, and origin) and the addition of zeolite. The results are inconclusive and indicate a need for further research, testing different doses of zeolite in different forms. Studies on the use of zeolite may help reduce the negative impact of poultry production on the environment, and ensure its safety and profitability.

**Abstract:**

The safety of production and the high quality of meat are important aspects of rearing poultry, especially when natural solutions are used. Because of the increasing popularity of duck meat, the aim of the present study was to assess the effect of a diet with an inclusion of zeolite on the growth performance, meat quality, and strength of the bones and jejunum in ducks of different origin, sex, and age at slaughter. The study was conducted on 320 Orvia and Cherry Valley ducks. Birds were allocated to eight groups, according to their sex and origin. Half of the birds received feed with a 4% inclusion of zeolite. Body weight gain, feed intake, and feed conversion ratio per kilogram of gain were calculated. After six or seven weeks, five birds from each group were selected and slaughtered. After dissection, meat quality (pH, water-holding capacity, colour) and the strength of the bones and gut were analysed. The analysis revealed that zeolite was associated with reduced body weight gains and increased feed conversion ratio, but with a higher water-holding capacity of breast muscles in seven-week-old ducks, and with a higher yellowness and water-holding capacity of leg muscles in six-week-old ducks. A positive effect of long-term factors (age, sex, origin) and the interaction with zeolite was found for most of the analysed traits. Findings on the effect of 4% inclusion of zeolite in duck diet were inconclusive. The study implies the need for further research, since zeolite has potential as a natural sanitizing agent and can improve the quality of produced duck meat.

## 1. Introduction

The dynamic growth and advances in poultry production bring benefits, but also challenges to growers associated with the cost-effectiveness of production as well as the quality and safety of meat [1]. Aluminosilicates, including zeolite, are becoming increasingly popular natural minerals that can influence the health of birds and other farm animals [2]. Zeolite is a crystalline mineral with three-dimensional pores that retain water without the modification of aluminosilicate chemical structure. In addition, zeolite actively adsorbs toxins (e.g., aflatoxin), carbon dioxide, hydrogen sulphide, and ammonia [3,4], which is the main secondary metabolite in poultry production, responsible for environmental pollution, and able to modify immune response [5,6].

In a study by Papaioannou et al. [7], natural and synthetic zeolites were mainly used in the diet of farm animals to improve their performance traits. Zeolite has been used as a feed additive for broiler chickens [8,9,10] and had positive effects on performance traits, and similar results were found in laying hens [11]. Previous studies have focused on the effect of different aluminosilicates, such as bentonite, kaolin, and zeolite, on meat quality in broiler chickens [10,12], including the chemical composition of meat [13]. Meat quality is determined by a number of parameters, such as the content of intramuscular fat, water-holding capacity, and colour, indicating the suitability of meat for processing and driving consumer choices [14].

Studies have also investigated the effect of a diet with zeolite on intestinal histology in ducks. Because the hypertrophy of intestinal villi and improved body weight gain were observed, researchers suggested that the maximum level of the supplement should be 1 g/kg of feed [15]. The jejunum is the middle part of the small intestine where the final digestive processes take place and nutrients are absorbed. Excessive levels of soluble non-starch polysaccharides may reduce the passage of food in the gut, immobilize digestive enzymes, and reduce the absorption of nutrients by the intestinal villi [16]. Feed additives stimulate the development of the intestines and the formation of the gut microbiome [17]. Banaszak et al. [10] reported a positive effect of halloysite on the development of intestinal villi. Feed formulation also affects the development of the gastrointestinal tract, intestinal peristalsis, and tensile strength of the gut (thickness of the muscular layer) [18]. Insoluble compounds, including minerals, improve intestinal peristalsis, and reduce feed density [19]. Wu et al. [20] reported the positive effect of a diet supplemented with zeolite on intestinal morphology. The effects of diet and feed additives on the tensile strength of chicken gut were investigated by Cowieson et al. [21]. They showed that feed additives may promote intestinal tensile strength but concluded that further research is needed.

Another important parameter in the production of broiler ducks is the quality of feet, including bones. Strong leg bones (femur, tibia, and fibula) help maintain the optimal welfare of birds. Bone strength depends, among other things, on the level of bioavailable calcium and phosphorus in the feed [22]. The absorption of these minerals can be improved by the addition of zeolite in feed.

Growth performance and generally defined meat quality also depend on long-term factors, which include the origin, sex, and age of birds at slaughter [23,24].

Nevertheless, reports on the use of natural aluminosilicates (zeolite) in poultry production are still limited. This research problem addresses innovative and relatively easy-to-implement solutions to ensure the safe production of broiler ducks and high quality of meat. Shariatmadari [3] indicated that the effects of zeolite have not been investigated in birds of different origin, age, and sex.

The tested hypothesis is as follows: A 4% inclusion of zeolite in feed influences the growth performance, carcass traits, and physicochemical parameters of breast and leg muscles, as well as the strength of the bones and jejunum in broiler ducks of different origin, sex, and age.

## 2. Materials and Methods

The study was carried out as part of pilot testing. Ducks were reared in conditions similar to those in commercial poultry farms. No approval was required according to Directive No. 2010/63/EU and Resolution No. 13/2016 of the Local Ethics Committee of 17 June 2016.

### 2.1. Duck Rearing

In the experiment, we used 320 one-day-old ducklings supplied from a hatchery (160 Cherry Valley ducks (English Pekin ducks) and 160 Orvia ducks (French Pekin ducks)). The birds were sexed (80 males and 80 females from both meat-type hybrids) and assigned to 8 groups, with 40 birds per group (5 replicates, 8 birds per group). In total, four groups of ducks received a commercially available loose feed, and the other four groups received feed supplemented with 4% of zeolite. Allocation to groups is presented in Table 1. Ducks were reared for 42 or 49 days.

Ducks were managed in line with the technology for the intensive production of broiler ducks in a building under controlled environmental conditions. The temperature inside the building at the beginning of the rearing period was 23 °C in the first week and then gradually reduced to 18 °C. In the first four weeks, additional sources of heat (radiators) were used, and the local temperature was 30 °C. The rate of air exchange was 1 m^3^/s. According to relevant standards for broiler duck production, the light availability was continuous (24 h) for 3 days, and then reduced to 16 h of light per day. The maximum stocking rate was 17 kg of live weight of birds per square meter. Ducks received feed and fresh drinking water ad libitum. Feeds were purchased from a manufacturer of feeds for waterfowl, and their basic analytical composition is presented in Table 2. Feeds were iso-protein and isocaloric, as were all ingredients provided (it was a commercial feed). According to the manufacturer, the starter feed contained wheat, soybean extraction meal, wheat bran, corn, sunflower seed extraction meal, wheat gluten, rapeseed extraction meal, calcium carbonate, animal (poultry) fat, 1-calcium phosphate, vegetable (soybean) oil, sodium chloride, and sodium bicarbonate, and vitamin A (10,000 IU/kg), vitamin D3 (3000 IU/kg), vitamin E (25 mg/kg), and a mixture of trace elements—manganese (II) oxide (70 mg/kg), iron (monohydrate 40 mg/kg), zinc (40 mg/kg), copper (8 mg/kg), iodine (0.8 mg/kg), and selenium (0.2 mg/kg)—were added. The grower contained wheat, wheat bran, corn, wheat gluten, triticale, soybean extraction meal, rapeseed and sunflower seed extraction meal, animal (poultry) fat, calcium carbonate, 1-calcium phosphate, and sodium chloride, and vitamin A (10,000 IU/kg), vitamin D3 (3000 IU/kg), vitamin E (25 mg/kg), and a mixture of trace elements: manganese (II) oxide (70 mg/kg), iron (monohydrate 40 mg/kg), zinc (40 mg/kg), copper (8 mg/kg), iodine (0.8 mg/kg), and selenium (0.2 mg/kg) were added. Both types of feed contained digestibility-enhancing substances (phytase and xynalases) and antioxidants (tocopherol extract from vegetable oils, butylated hydroxytoluene, and butylated hydroxyanisole). Experimental feed was supplemented with 4% of zeolite (Clinoptilolite). Feed was supplied to birds as a meal. The zeolite delivered from the supplier was in a loose (dusty) form. It was a hydrated aluminosilicate of alkali metals and alkaline earth metals. It was mixed with the feed, which also had a loose form, which allowed for the uniformity of the feed structure and made sure that the zeolite did not reach the bottom of the feeders separately. According to the supplier’s characteristics, the natural zeolite used had a specific surface area of 30–60 m^2^/g, a bulk density of 1.60–1.80 kg/m^3^, and a specific weight of 2.20–2.44 kg/m^3^. The main component of the zeolite used was silicon dioxide (SiO_2_) at the level of 65–71.30% and aluminium oxide (Al_2_O_3_) at 11.50–13.10%. In addition, the chemical composition was shown to contain calcium oxide (CaO, 2.70–5.20%), potassium oxide (K_2_O, 2.20–3.40%), iron (III) oxide (Fe_2_O_3_, 0.70–1.90%), magnesium oxide (MgO, 0.60–1.20%), sodium oxide (Na_2_O, 0.20–1.30%), titanium oxide (TiO_2_, 0.10–0.30%), and the elements silicon (Si), and aluminium (Al) at the level 4.80–5.40% of Si/Al. Zeolite also contained minerals such as clinoptilolite (84%), cristobalite (8%), mica clay (4%), plagioclase (3–4%), rutile (0.10–0.30%), and traces of quartz. In line with the technology of broiler duck production, there were two feeding phases when ducks were on a starter diet (Days 0–21) and a grower diet (Days 22–42 or 22–49). The presented research was of a pilot nature, and zeolite in duck rearing was tested as an innovation. A zeolite addition of 4% was considered the correct maximum level based on a market study for zeolite products. Most producers and suppliers recommended the addition of zeolite in poultry feed (mainly chicken broilers and layers) to a maximum of 4%. No information was found regarding such use of aluminosilicate in the rearing of ducks, so the decision on a 4% addition of zeolite to the feed in the experiment was made to determine or exclude the validity of using such an amount of the mineral.

### 2.2. Growth Performance

During rearing, the ducks were weighed on three dates: Day 0 (stocking), Day 21 (feed change), and Day 42 or Day 49 (slaughter). Feed intake (FI) was recorded daily, and records were used for the calculation of mean body weight gain (BWG) and the feed conversion ratio (FCR).

### 2.3. Slaughter, Carcass Traits, and Meat Quality

The birds were slaughtered on two dates: Day 42 or 49 of rearing. The slaughter of birds after 6 weeks was done to shorten the common rearing period. The material of commercial hybrids makes it possible to rear effectively for 6 weeks. Overall, 5 birds from each group (one per replicate; 40 birds per date, 80 ducks in total) were randomly selected. Selected ducks were weighed, stunned by means of an electric current, and decapitated at the atlanto-occipital joint. This allowed for rapid exsanguination. Rapid bleeding after stunning the birds is a humane method that reduces suffering and is carried out in accordance with the applicable legislation on the protection of animals at the time of killing. The carcasses were scalded in a 65 °C water bath to loosen and remove feathers. Carcasses were further cleaned by waxing to remove feather residues (quills). Feet were cut off at the ankle joints and carcasses were gutted. Gutted carcasses and edible offal (heart, gizzard, and liver) were kept for further analyses. A fragment of ileum was also dissected (Section 2.4).

The pH value of breast muscles (*pectoralis major*) was measured 45 min post-mortem (pH_45m_) using a pH-meter (Elmetron, Zabrze, Poland) with a knife electrode. Carcasses prepared for analysis were chilled in a cold room (Hendi, Poznań, Poland) at 4 °C for 24 h. Chilled carcasses were weighed (Radwag, Radom, Poland) and measured again for the pH of muscles (pH_24h_). Each carcass was dissected, following a procedure described by Banaszak et al. [25], by separating the neck, wings with skin, skin with subcutaneous fat, breast muscles, leg muscles without bones, abdominal fat, and carcass remains (trunk and leg bones). All elements were weighed, and their proportions in the carcass were calculated ($\frac{weight\;of\;element}{weight\;of\;carcass}\times \;100\%$). Dressing percentage was calculated from the formula ($\frac{carcass\;weight}{live\;body\;weight}\;\times \;100\%$). The right leg bones were kept for further analyses (Section 2.4).

Breast and leg muscles (right) were analysed for colour (Konica Minolta colorimeter, Tokyo, Japan) in the CIE L*a*b* system (Comission Internationale de l’Eclairage). The colour was defined using the parameters of lightness (L*), redness (a*), and yellowness (b*) measured on the outer side of the muscles. After the analysis of colour, the muscles were tested for drip loss. Each muscle was weighed (M1) and then placed in two bags, the inner of which was perforated at the bottom. Samples were left to hang in a cold room at 4 °C for 24 h and weighed again (M2), and the drip loss from meat was calculated in percent from the formula $\left(100-\left(\frac{M1}{M2}\right)\right)\times \;100\%$. Breast and leg muscles (left) were analysed for water-holding capacity. Portions of meat from treatment groups were disintegrated in a mincer (Hendi, Poznań, Poland), 0.295–0.305 g samples were prepared (M1), placed between two pieces of Whatman 1 filter paper, and kept under 2 kg pressure for 5 min. After 5 min, samples were reweighed (M2). Water loss from meat (in %) was calculated from the difference between M1 and M2. In addition, 90 g samples of minced breast and leg muscles were prepared and analysed for their chemical composition (content of protein, collagen, sodium chloride, and intramuscular fat and water). Analyses were performed using the FoodScan apparatus (FOSS, Hilleroed, Denmark) and Near InfraRed Transmission (NIT) spectrometry. Water-holding capacity and chemical composition were analysed in 5 replicates for each treatment group. Procedures for the analysis of physicochemical traits of breast and leg muscles were consistent with descriptions provided by Biesek et al. [26] with minor modifications.

### 2.4. Bones’ Breaking Strength and Jejunum Tensile Strength

The trimmed right tibia from each duck’s leg was used for the analysis of breaking strength. Jejunum tensile strength was also analysed. Gut samples were collected immediately after slaughter. A fragment of the jejunum from Meckel’s diverticulum to the transition point of the jejunum into duodenum was dissected. Samples were frozen for 48 h, thawed at 4 °C, and analysed for tensile strength using an Instron 3345 apparatus (Instron, Buckinghamshire, UK) integrated with Bluehill 3 software.

The bone strength was analysed using a Instron Bend Fixture 10 mm Anvil adapter. Tibial bones were placed between the clamps, and the maximum load and force at breaking (N) and the deformation in response to compressing force and dislocation (mm) were measured. Measurements were taken at a rate of 250 mm/min. Methods were described by Kuźniacka et al. [27].

Jejunum tensile strength was assessed based on the measured maximum force at breaking (N). The load applied to the jejunum was simulated using a Instron Pneumatic Grip 2kN adapter. Standardized gut samples (each 5 cm long) were placed between two adapters and stretched. Gut samples were standardized with respect to Meckel’s diverticulum. Measurements were taken at a rate of 500 mm/min. Jejunum samples were stretched according to a procedure described by Budnik [28].

### 2.5. Statistical Analysis

Numerical data were processed with Statistica software 13.0 (2017, Statsoft, Kraków, Poland). Growth performance parameters were presented as descriptive statistics, and the significance of differences was tested with a simple ANOVA procedure. Data for each analysed factor were verified with the Student t-test at the significance level of *p* < 0.05. Other calculations were performed using a multivariate analysis of variance (ANOVA) in which four grouping factors were considered (age, origin, sex, and zeolite). The mean values of the analysed variables were calculated for the two age groups (6 and 7 weeks) and for the control and treatment groups (zeolite). The standard error of the mean (SEM) for all effects combined was calculated using descriptive statistics. Statistically significant differences were verified using the post–hoc Sheffe test at the significance level of *p* < 0.05. We also analysed the significance of differences for each factor alone and the interaction zeolite x long-term factors by verifying one-dimensional results for each dependent variable.

The calculations made in the presented research work are the result of rearing. The main element of this research was the use of zeolite in duck feed. Long-term factors (age, sex, and origin) were additional elements that could be demonstrated (to deepen knowledge), so their results and interactions were presented only as significance values, not as mean values.

Each value in the production results was calculated from replications (pens) indicated in the duck rearing section, while the laboratory analyses for meat quality and the strength of the bones and jejunum were calculated from the number of ducks selected for slaughter (*n* = 8), as also described in the duck rearing section. In qualitative research, each bird and its elements constituted the basic experimental unit.

## 3. Results

The mortality rate in the flock did not exceed 2%, and deaths were related to the culling of the weak ducklings, which was recorded in the first days of rearing.

### 3.1. Growth Performance

The analysis of data presented in Table 3 revealed a significantly higher weight gain in ducks on a diet without zeolite on Days 0–21 and 22–49, as well as during the whole six- or seven-week rearing period (*p* < 0.05). In addition, feed intake in this group was significantly higher compared to control ducks on a diet with zeolite (*p* < 0.05), except for the whole seven-week rearing period (Days 0–49). The feed conversion ratio per kg of weight gain (FCR) was significantly higher in the group of ducks on a diet with zeolite in the first period of rearing (Days 0–21), and this resulted in a significant difference for the whole period of 0–49 days, where FCR was lower in ducks on a diet without zeolite (*p* < 0.05). There were no significant differences in terms of the origin and sex of birds (*p* > 0.05). Data analysis did not reveal significant interactions between the factors (*p* > 0.05), which is reflected by the values presented in Table 3.

### 3.2. Carcass Traits

Table 4 and Table 5 show results of the carcass composition and the content of muscles and fat. In six-week-old ducks on a diet without zeolite, only a significantly higher proportion of the liver in carcass was found, compared to birds on a diet with zeolite (*p* = 0.044). There were no significant differences between other carcass traits (*p* > 0.05).

In seven-week-old ducks, body weight and carcass weight were significantly higher in the groups without zeolite compared to other treatment groups, where the feed was supplemented with 4% zeolite (*p* = 0.011 and *p* = 0.044, respectively). Dressing percentage in the control and treatment groups was similar (*p* = 0.837). There were no significant differences in other traits between seven-week-old ducks (*p* > 0.05).

The analysis of one-dimensional results for carcass traits and the interactions between the grouping factors demonstrated the influence of the addition of zeolite, age, origin, and sex on the body weight of ducks. However, only age and sex had a significant effect on carcass weight (*p* < 0.001). There was a significant interaction between zeolite and age for both traits, an interaction between zeolite and other long-term factors, and a three-factor interaction of age*origin*zeolite and origin*sex*zeolite for the body weight of ducks. Dressing percentage was significantly influenced by age (*p* < 0.001) and sex (*p* = 0.047). Results present many statistically significant relationships between factors. In particular, the age at slaughter, origin, and sex had a significant effect on the proportion of breast muscles in carcass (independently), while the proportion of leg muscles depended only on age. The total proportion of muscles in the carcass depended significantly on all factors separately, without any significant interaction between them. Only the proportion of the liver in carcass depended on the interaction between zeolite and age at slaughter (*p* = 0.009), while the proportion of the heart depended on the interaction of all factors (*p* = 0.02).

### 3.3. Breast and Leg Muscle Quality

Table 6 and Table 7 present data on the physicochemical parameters of breast and leg muscles important for meat quality. The ability of breast and leg muscles to retain water, expressed as the water-holding capacity, was significantly better in six-week-old ducks on a diet without zeolite (*p* = 0.012; <0.001, respectively). However, in seven-week-old ducks, the loss of water for breast muscles from ducks on a diet with zeolite was lower (*p* = 0.049). Yellowness (b*) of leg muscles from ducks on a diet with zeolite was significantly higher than in the control groups (I–IV). The content of protein (*p* = 0.019) and water (*p* < 0.001) in breast muscles from six-week-old ducks was significantly higher in the control groups than in the treatment groups (V–VIII). The content of protein was also significantly higher in leg muscles from ducks on a diet without zeolite (*p* < 0.001). After seven weeks of rearing, in addition to significant differences in the water-holding capacity (WHC) of breast muscles, there was a significantly greater proportion of intramuscular fat in ducks fed a diet with zeolite (*p* = 0.002). No significant differences were found between other analysed traits (*p* > 0.001).

Statistical analysis of data on the quality traits of breast muscles (Table 6) and leg muscles (Table 7) revealed significant one-dimensional relationships for all experimental factors. We observed an effect of age and sex on the pH value of muscles 45 min post-mortem (pH_45min_) and an effect of age on pH_24h_. For breast muscles, there was a significant effect of age and sex on lightness (L*) and one of the interaction of zeolite and age on redness (*p* = 0.042). Yellowness depended on the interaction between zeolite and sex (*p* = 0.018). Age and its interaction with zeolite, as well as the interaction zeolite*origin had a significant effect on the water-holding capacity of breast muscles. All factors (zeolite and long-term factors) and their interactions in different combinations influenced the content of protein in breast muscles. A similar effect was found for the content of intramuscular fat (except for origin*sex*zeolite), and the content of water (except for age*sex*zeolite). The content of collagen in breast muscles was influenced by age, sex, and the zeolite*origin, zeolite*sex, age*origin*zeolite, and origin*sex*zeolite interactions, and all four factors combined. Considering the one-dimensional results, there was a significant dependence between age and age*origin*zeolite, between lightness (L*), age, and parameter a*, and between zeolite and parameter b* for leg muscles (*p* < 0.05). Zeolite, age, and sex, the zeolite*age, zeolite*sex, and origin*sex*zeolite interactions, and all factors combined had a significant effect on the water-holding capacity of leg muscles. Levels of basic chemical components in leg muscles also depended on zeolite supplementation and long-term factors and their interaction in different combinations, especially with respect to intramuscular fat, for which one-dimensional analysis showed the effect of all factors in each combination (*p* < 0.05), and the content of water, except zeolite supplementation. All factors had a significant effect on the content of protein in breast muscles, except for the zeolite*origin interaction (*p* > 0.05). The content of collagen depended on sex and the interaction between zeolite and long-term factors, except for the interactions of origin*sex*zeolite and all factors combined.

### 3.4. Bones’ Breaking Strength and Jejunum Tensile Strength

Table 8 shows data on the breaking strength of tibial bones from ducks, expressed as the maximum load, load at breaking, and compressive deformation. The analysis demonstrated the effect of age on the maximum load and load at breaking, while the compressive deformation (gauge length) correlated with sex and age, as well as the interactions age*sex*zeolite and origin*sex*zeolite (*p* < 0.05). No significant differences between the groups and interactions between the factors in terms of bones’ breaking strength were found for other parameters (*p*> 0.05). Similarly, no significant differences or interactions between factors were found with respect to jejunum tensile strength, expressed as the maximum load and tensile dislocation (*p* > 0.05). Mean values were higher in the group of ducks on a diet containing zeolite (15.90 N in six-week-old ducks and 16.75 N in seven-week-old ducks) compared to the ducks from the control group (14.63 N; 16.30 N, respectively), but the differences were not significant.

## 4. Discussion

Shariatmadari [3] presented a review of studies on the use of zeolite in poultry production. The differences in results, either positive or negative, were attributed to different levels of zeolite in feed, which may affect the concentration of various nutrients and feed consistency. Shariatmadari [5] also addressed the issue of body weight gain. Our study found significantly lower body weight gains and higher feed intakes in ducks reared for 6 weeks, which may prove an excessive zeolite level in duck feed. This can be compared to findings by Khambualai et al. [15], who reported a higher body weight gain in nine-week-old Aigamo ducks on a diet supplemented with zeolite and plant extracts at a level from 0.1 to 0.5 g/kg of feed. Body weight and carcass weight were also lower in ducks receiving zeolite. Other researchers [29] investigated the effect of 2% of zeolite in the diet of laying ducks and reported a greater body weight in these birds, which again implies that the excessive supplementation of zeolite may have a negative effect on the body weight of birds. Chung and Choi [30] demonstrated that a diet with a 2% inclusion of bentonite had no effect on the body weight of ducks, but a higher feed conversion ratio was reported, similar to our study.

A diet with a 2% inclusion of natural or modified zeolite had no significant effect on the growth performance of broiler chicken [20]. On the other hand, Christaki et al. [31] investigated the effect of a diet supplemented with zeolite (2%) and flaxseed (3 or 10%) on the performance of chicken. They reported a higher weight of leg muscles and a reduced proportion of abdominal fat in the carcass, which suggests a positive effect of natural zeolite on the deposition of fatty tissue in birds. Our research did not reveal significant differences in the content of abdominal fat, but the mean values in the six-week-old ducks were lower in the group on a diet with zeolite. Our study demonstrated a relationship between the proportion of leg muscles in the carcass and the age of ducks (*p* < 0.001). Zeolite at a dose of 5 g/kg of feed had no effect on the dressing percentage of broiler chicken carcasses, as reported by Schneider et al. [32]. Similar findings were made in our study, but the dressing percentage depended on the age and sex of ducks. Preyavitchayapugdee and Prapasanobola [33] reported that a 3% inclusion of zeolite was associated with a significantly lower weight of wings in broiler ducks. Our study demonstrated a relationship between the proportion of wings in carcass and age, and the interaction between age, origin, and zeolite. We also found a lower proportion of the liver in six-week-old ducks. The liver has metabolic and digestive functions [34]. It can be assumed that the size of this organ could depend on the addition of zeolite, which affects the availability of nutrients (as mentioned earlier in the presented paper).

In our study, we demonstrated the positive effect of zeolite on the water-holding capacity of breast muscles from seven-week-old ducks. We also observed the effect of age, as well as the interactions between age and zeolite and between origin and zeolite. The water-holding capacity of breast muscles depends on the content of muscle fibres involved in oxidation [35]. WHC is also affected by proteolysis in breast muscles [36]. In our study, drip loss from breast and leg muscles was significantly higher in six-week-old ducks, but we also found a lower content of protein in muscles, which could be associated with poorer feed conversion. Univariate analysis showed that the water-holding capacity of leg muscles depended on the addition of zeolite, age, sex, and the interaction between long-term factors and zeolite. On the other hand, the content of protein in breast and leg muscles depended on all the analysed factors and interactions between them. The content of intramuscular fat and the chemical composition of breast and leg muscles depend on the origin (genotype), age, sex, and diet of birds [37].

Bone strength is an important parameter reflecting the correct production and welfare of birds. The results of previous studies indicated the positive effect of zeolite on bone strength associated with calcium and phosphorus levels, and the availability of these elements should be considered [5]. Shariatmadari noted that zeolite may increase the utilization of calcium, but there is a concern about decreased phosphorus availability. Our study revealed that zeolite had no negative effect on bone strength, but we observed the effect of age, which was also reported by Rath et al. [38].

Bilgili and Hess [39] found that the tensile strength of broiler intestines increased with age and was higher in male than in female chickens. Our study did not reveal any dependence between analysed variables with respect to the maximum load at which the jejunum is ruptured. The lack of significant differences may be associated with the fact that the flock of birds was homogeneous regardless of sex. It is related to significant advances in breeding, where restrictive selection affects many traits of ducks [40]. Zeolite may influence gut morphology (the size and shape of the intestinal villi), the intestinal microbiota, and the digestibility of proteins and increase the levels of the apparent metabolizable energy (AME; AME_N_) [3]. For example, Banaszak et al. [10] demonstrated a positive effect of aluminosilicates on the histomorphometric traits of the jejunum in broiler chickens. Nevertheless, data on the tensile strength of the intestines, including the effect of zeolite in feed on this trait, are limited.

## 5. Conclusions

A diet with a 4% inclusion of zeolite (maximum dose recommended for poultry according to different producers) had no negative effect on most of the examined carcass traits, the bone or gut strength, or the dressing percentage of male and female Orvia and Cherry Valley broiler ducks reared for six or seven weeks. The addition of zeolite had a positive effect on the water-holding capacity of breast muscles from seven-week-old ducks, as well as the water-holding capacity and yellowness of leg muscles in six-week-old ducks. The study demonstrated many interactions between the use of zeolite and long-term factors, i.e., the age at slaughter, sex, and the origin of birds. The obtained results raise many questions that would be worth answering. A 4% inclusion of zeolite in duck feed reduced growth performance parameters, but other researchers reported that lower doses of zeolite in chicken feed improve growth performance and meat quality. Further studies are required to investigate different (lower) levels of zeolite in the feed, and the effect of a diet with a different form of zeolite should be tested in ducks. Findings from the presented study are inconclusive, but there are many evidence-based recommendations that indicate substantial benefits from the use of aluminosilicates as agents, contributing to a cleaner environment and improving production performance and meat quality.

## Figures and Tables

**Table 1:** Description of experimental groups of ducks.

Group No.	Treatment	Origin	Sex	Content of Zeolite in Feed
I	Control	Orvia	male	-
II		Cherry Valley	female	-
III		Orvia	female	-
IV		Cherry Valley	male	-
V	Zeolite	Orvia	male	4%
VI		Cherry Valley	female	4%
VII		Cherry Valley	male	4%
VIII		Orvia	female	4%

**Table 2:** Analytical composition of feeds for broiler ducks.

	Control		Zeolite	
Ingredients (%)	Starter	Grower	Starter	Grower
Dry matter	88.35	87.05	88.22	87.38
Crude ash	4.78	4.67	4.91	4.86
Crude protein	19.07	16.26	18.54	15.74
Crude fat	3.94	4.22	3.53	3.67
Crude fibre	4.60	4.57	4.87	4.59
Starch	38.63	41.80	38.18	40.91
Lysine	0.93	0.84	0.93	0.84
Phosphorus	0.65	0.61	0.65	0.61
Calcium	0,64	0.60	0,64	0.60
Threonine	0.66	0.58	0.66	0.58
Methionine	0.42	0.36	0.42	0.36
Tryptophane	0.25	0.22	0.25	0.22
Sodium	0.16	0.15	0.16	0.15

**Table 3:** Growth performance of broiler ducks.

Days	Control	Zeolite	Orvia	Cherry Valley	Male	Female	SEM	*p*–Value		
								Zeolite	Origin	Sex
BWG, g										
0–21	1138 ^a^	1024 ^b^	1089	1074	1069	1093	22.87	<0.001	0.774	0.626
22–42	1852	1742	1760	1833	1764	1829	34.02	0.123	0.339	0.388
22–49	2482 ^a^	2254 ^b^	2310	2426	2370	2365	57.67	0.033	0.354	0.969
0–42	2990 ^a^	2767 ^b^	2849	2907	2833	2923	50.95	0.011	0.611	0.416
0–49	3621 ^a^	3278 ^b^	3398	3499	3439	3459	74.16	0.005	0.538	0.905
FI, g										
0–21	2183 ^a^	2052 ^b^	2143	2091	2075	2159	34.38	0.043	0.494	0.252
22–42	7299 ^a^	7080 ^b^	7193	7188	7180	7199	43.41	<0.001	0.959	0.851
22–49	10,078 ^a^	9821 ^b^	9953	9946	9941	9957	51.02	<0.001	0.952	0.889
0–42	9482 ^a^	9132 ^b^	9336	9279	9256	9358	73.54	0.002	0.730	0.531
0–49	14,338	14,640	14,144	14,834	14,694	14,284	218.01	0.532	0.117	0.387
FCR, kg/kg										
0–21	1.92^b^	2.00 ^a^	1.97	1.95	1.95	1.98	0.02	0.039	0.644	0.540
22–42	3.94	4.08	4.10	3.92	4.09	3.93	0.08	0.421	0.276	0.369
22–49	4.07	4.37	4.32	4.11	4.20	4.23	0.09	0.100	0.276	0.900
0–42	3.17	3.31	3.28	3.19	3.27	3.20	0.04	0.141	0.339	0.468
0–49	3.96 ^b^	4.46 ^a^	4.17	4.25	4.28	4.15	0.12	0.017	0.776	0.633

^a^,^b^: means in the same line with no common superscript differ between groups (control: zeolite) within weeks (*p* < 0.05); SEM: standard error of the mean for all of data; BWG: body weight gain, g; FI: feed intake, g; FCR: feed conversion ratio, kg/kg.

**Table 4:** Body weight and carcass traits in broiler ducks *.

Group	g		%	% in Carcass of					
	Body Weight	Carcass Weight	Carcass Yield	Wings	Neck with Skin	Remaining Parts	Heart	Liver	Gizzard
	6 Weeks								
Control	2922.45	1994.07	68.22	13.02	13.67	29.81	0.76	3.23 ^a^	3.80
Zeolite	2911.85	1998.98	68.63	12.69	13.80	28.15	0.78	2.90 ^b^	3.86
*p*-value	0.990	0.999	0.923	0.854	0.996	0.582	0.859	0.044	0.991
	7 Weeks								
Control	3537.45 ^a^	2483.00 ^a^	70.15	12.30	12.78	27.54	0.77	2.74	3.38
Zeolite	3427.40 ^b^	2385.19 ^b^	69.60	12.00	13.35	27.18	0.74	2.84	3.66
*p*-value	0.011	0.044	0.837	0.892	0.758	0.992	0.777	0.856	0.460
SEM	33.96	27.51	0.23	0.14	0.19	0.43	0.02	0.05	0.07
	*p*-value and interaction between zeolite and long-term factors (one-dimensional results)								
Zeolite	<0.001	0.339	0.867	0.232	0.329	0.212	0.898	0.153	0.111
Age	<0.001	<0.001	<0.001	0.009	0.065	0.048	0.410	0.001	0.005
Origin	<0.001	0.303	0.149	0.382	0.760	0.153	0.959	0.340	0.236
Sex	0.022	0.034	0.047	0.927	0.005	0.012	0.239	0.044	<0.001
Zeolite*Age	0.004	0.036	0.239	0.944	0.538	0.420	0.173	0.009	0.286
Zeolite*Origin	0.045	0.876	0.247	0.613	0.866	0.578	0.922	0.563	0.175
Zeolite*Sex	0.021	0.222	0.987	0.139	0.805	0.546	0.413	0.660	0.018
Age*Origin*Zeolite	1.000	0.596	0.333	0.048	0.973	0.495	0.389	0.846	0.201
Age*Sex*Zeolite	0.002	0.203	0.073	0.463	0.292	0.157	0.959	0.209	0.309
Origin*Sex*Zeolite	0.011	0.574	0.657	0.516	0.444	0.085	0.486	0.426	0.265
Zeolite*Age*Origin*Sex	0.084	0.101	0.079	0.080	0.061	0.844	0.020	0.233	0.828

^a^,^b^: means in the same column with no common superscript differ between groups (control: zeolite) within weeks (*p* < 0.05); SEM: standard error of the mean for all data; * results for ducks selected for slaughter.

**Table 5:** Muscle and fat content in broiler ducks carcasses *.

Group	% in Carcass of				
	Breast Muscles	Leg Muscles	Total Muscles	Skin with Subcutaneous Fat	Abdominal Fat
	6 Weeks				
Control	13.27	14.69	27.96	20.15	0.81
Zeolite	14.86	15.30	30.17	19.89	0.56
*p*-value	0.237	0.644	0.082	0.991	0.585
	7 Weeks				
Control	18.66	13.39	32.05	19.85	0.62
Zeolite	18.76	13.72	32.48	19.11	0.72
*p*-value	0.999	0.923	0.966	0.830	0.962
SEM	0.38	0.18	0.35	0.28	0.06
	*p*-value and interaction between zeolite and long-term factors (one-dimensional results)				
Zeolite	0.072	0.176	0.016	0.367	0.538
Age	<0.001	<0.001	<0.001	0.315	0.926
Origin	<0.001	0.348	<0.001	0.075	0.058
Sex	<0.001	0.172	0.012	0.006	0.101
Zeolite*Age	0.114	0.176	0.101	0.647	0.167
Zeolite*Origin	1.000	0.679	0.894	0.861	0.591
Zeolite*Sex	0.355	0.836	0.161	0.038	0.276
Age*Origin*Zeolite	0.113	0.188	0.587	0.314	0.054
Age*Sex*Zeolite	0.524	0.630	0.806	0.455	0.558
Origin*Sex*Zeolite	0.139	0.941	0.181	0.236	0.262
Zeolite*Age*Origin*Sex	0.695	0.905	0.791	0.456	0.551

means in the columns do not differ between groups (control: zeolite) within weeks (*p* > 0.05); SEM: standard error of the mean for all data; * results for ducks selected for slaughter.

**Table 6:** Physicochemical parameters of breast muscles from broiler ducks *.

Group	pH		Colour			%					
	45 min	24 h	L*	a*	b*	Drip Loss	WHC	Protein	Collagen	Fat	Water
	6 Weeks										
Control	6.41	6.38	48.36	10.80	3.17	0.84	27.96 ^b^	21.13 ^a^	1.33	2.35	77.78 ^a^
Zeolite	6.47	6.38	47.89	11.57	3.57	0.69	33.15 ^a^	20.69 ^b^	1.26	2.24	78.45 ^b^
*p*-value	0.907	0.996	0.944	0.532	0.895	0.890	0.012	0.019	0.407	0.860	<0.001
	7 Weeks										
Control	6.55	6.02	45.81	11.61	3.02	0.52	40.15 ^a^	21.10	1.19	2.58	76.98
Zeolite	6.65	6.27	46.28	10.91	2.30	0.55	35.81 ^b^	21.11	1.17	2.61	76.87
*p*-value	0.837	0.378	0.941	0.612	0.599	0.469	0.049	0.999	0.975	0.996	0.832
SEM	0.04	0.05	0.28	0.18	0.19	0.58	0.72	0.05	0.02	0.05	0.08
	*p*-value and interaction between zeolite and long-term factors (one-dimensional results)										
Zeolite	0.237	0.292	0.993	0.919	0.669	0.637	0.621	<0.001	0.018	<0.001	<0.001
Age	0.033	0.021	<0.001	0.837	0.056	0.081	<0.001	<0.001	<0.001	<0.001	<0.001
Origin	0.979	0.592	0.607	0.077	0.876	0.140	0.276	<0.001	0.826	<0.001	<0.001
Sex	0.033	0.583	<0.001	0.121	0.150	0.072	0.206	<0.001	<0.001	<0.001	<0.001
Zeolite*Age	0.898	0.171	0.331	0.042	0.126	0.526	<0.001	<0.001	0.166	<0.001	<0.001
Zeolite*Origin	0.405	0.271	0.246	0.638	0.383	0.899	0.002	<0.001	0.017	<0.001	<0.001
Zeolite*Sex	0.525	0.447	0.381	0.355	0.018	0.594	0.398	<0.001	<0.001	<0.001	0.002
Age*Origin*Zeolite	0.379	0.376	0.535	0.482	0.901	0.664	0.125	<0.001	0.003	<0.001	<0.001
Age*Sex*Zeolite	0.979	0.613	0.804	0.286	0.935	0.313	0.065	<0.001	0.413	<0.001	0.283
Origin*Sex*Zeolite	0.653	0.448	0.046	0.807	0.498	0.184	0.165	<0.001	0.005	0.762	<0.001
Zeolite*Age*Origin*Sex	0.186	0.363	0.305	0.036	0.701	0.825	0.275	<0.001	<0.001	<0.001	0.001

^a^,^b^: means in the same column with no common superscript differ between groups (control: zeolite) within weeks (*p* < 0.05); SEM: standard error of the mean; L*: lightness; a*: redness; b*: yellowness; WHC: water-holding capacity; * results for ducks selected for slaughter.

**Table 7:** Physicochemical parameters of leg muscles from broiler ducks *.

Group	Colour			%				
	L*	a*	b*	WHC	Protein	Collagen	Fat	Water
	6 Weeks							
Control	43.65	9.21	0.96 ^b^	26.19 ^b^	19.49 ^a^	1.40	5.69	74.43
Zeolite	41.67	9.94	1.41 ^a^	34.66 ^a^	18.97 ^b^	1.29	5.73	74.86
*p*-value	0.287	0.689	0.044	<0.001	<0.001	0.055	0.995	0.076
	7 Weeks							
Control	40.33	10.42	0.98	39.60	19.31	1.34	5.10 ^b^	75.21
Zeolite	40.19	10.97	1.41	38.37	19.00	1.39	5.78 ^a^	74.83
*p*-value	0.999	0.844	0.965	0.717	0.066	0.587	0.002	0.136
SEM	0.38	0.22	0.30	0.69	0.05	0.02	0.07	0.06
	*p*-value and interaction between zeolite and long-term factors (one-dimensional results)							
Zeolite	0.128	0.144	0.023	<0.001	<0.001	0.208	<0.001	0.056
Age	<0.001	0.012	0.107	<0.001	<0.001	0.385	<0.001	<0.001
Origin	0.391	0.171	0.490	0.131	<0.001	0.142	<0.001	<0.001
Sex	0.697	0.943	0.924	0.020	<0.001	0.004	<0.001	<0.001
Zeolite*Age	0.183	0.830	0.107	<0.001	<0.001	<0.001	<0.001	<0.001
Zeolite*Origin	0.329	0.780	0.730	0.409	0.192	0.038	<0.001	<0.001
Zeolite*Sex	0.085	0.545	0.273	0.031	<0.001	0.005	<0.001	<0.001
Age*Origin*Zeolite	0.017	0.198	0.199	0.857	<0.001	<0.001	<0.001	<0.001
Age*Sex*Zeolite	0.909	0.899	0.943	0.116	<0.001	0.036	<0.001	<0.001
Origin*Sex*Zeolite	0.823	0.737	0.572	0.001	<0.001	0.312	<0.001	<0.001
Zeolite*Age*Origin*Sex	0.759	0.169	0.812	0.020	<0.001	0.907	<0.001	<0.001

^a^,^b^: means in the same column with no common superscript differ between groups (control: zeolite) within weeks (*p* < 0.05); SEM: standard error of the mean; L*: lightness; a*: redness; b*: yellowness; WHC: water-holidng capacity; * results for ducks selected for slaughter.

**Table 8:** Bones’ breaking strength and jejunum tensile strength of broiler ducks.

Group	Tibial Bones				Small Intestine (Jejunum)	
	Maximum Load [N/mm]	Load at Breaking (Stand.) [N]	Compressive Deformation (Dislocation) at Breaking (Cursor) [mm/mm]	Compressive Deformation (Dislocation) Gauge Length [mm]	Maximum Load [N]	Dislocation during Stretching [mm]
6 weeks						
Control	258.24	198.03	0.041	74.04	14.63	9.86
Zeolite	255.31	215.55	0.043	74.10	15.90	10.26
*p*-value ^×^	0.996	0.865	0.800	0.998	0.819	0.944
7 weeks						
Control	281.73	251.36	0.041	73.85	16.30	10.26
Zeolite	268.06	228.43	0.040	74.10	16.75	10.55
*p*-value ^×^	0.715	0.741	0.987	0.878	0.990	0.978
SEM	4.22	7.43	0.001	0.11	0.47	0.23
*p*-value and interaction between zeolite and long-term factors (one-dimensional results)						
Zeolite	0.324	0.841	0.663	0.457	0.373	0.472
Age	0.034	0.016	0.315	0.652	0.191	0.472
Origin	0.731	0.400	0.874	0.136	0.429	0.756
Sex	0.821	0.472	0.235	0.042	0.649	0.837
Zeolite*Age	0.523	0.137	0.351	0.634	0.666	0.905
Zeolite*Origin	0.204	0.118	0.561	0.411	0.202	0.362
Zeolite*Sex	0.840	0.767	0.426	0.116	0.926	0.905
Age*Origin*Zeolite	0.552	0.684	0.249	0.953	0.991	0.888
Age*Sex*Zeolite	0.928	0.457	0.301	0.022	0.102	0.707
Origin*Sex*Zeolite	0.167	0.177	0.936	0.039	0.839	0.318
Zeolite*Age*Origin*Sex	0.453	0.089	0.459	0.900	0.746	0.957

^×^: means in the columns do not differ between groups (control: zeolite) within weeks (*p* > 0.05); SEM: standard error of the mean for all data.

## Data Availability

All data, methods, and results of statistical analyses are reported in this paper. We remain at your disposal, in case of any questions.
